# Improving knowledge and attitudes about prevention and cessation of smoking using comics: study on youth with smoker’s social environment

**DOI:** 10.11604/pamj.2022.43.158.17821

**Published:** 2022-11-25

**Authors:** Abduh Ridha, Deni Ramadhani, Elly Trisnawati, Diah Radiana, Ufi Ruhama

**Affiliations:** 1 Faculty of Health Sciences, Universitas Muhammadiyah Pontianak, Pontianak, Indonesia,; 2 Public Health Office, Pontianak City, Indonesia

**Keywords:** Comics, health education, smoking cessation, adolescence, social environments

## Abstract

**Introduction:**

it is needed the innovative interventions to promote smoking cessation behavior. This research analyzed the influence of comic as the communication media to the knowledge and attitude of the respondent in the various determinant of smoking behavior.

**Methods:**

this research measured the knowledge and attitude of the respondent after they have been given comic as the educational media. It also explored the information about smoking status and social context of the smokers, such as smoker parents, friends, and teachers. The population was the students. The sample size was 1000 students of junior high school. The research aimed to find out the difference of knowledge and attitude based on the smoking status, social context, and training experience.

**Results:**

the mean score of knowledge between the respondents who had trained was higher than respondents who had no trained was significantly different (p<0.05). There was also significantly different of the mean score of knowledge and attitude (p<0.05) between the respondents whose family were smoker and non-smoker. The mean score of knowledge and attitude between the smoker and non-smoker respondents was also significantly different (p<0.05).

**Conclusion:**

the comic is more effective as an educational media for non-smoker adolescents, adolescents who have not smoker family, and health educated adolescent.

## Introduction

Smoking is the largest preventable cause of death in the world [1]. It is nearly 6 million people die every year due to tobacco use [2]. Tobacco can kill 8 million people per year worldwide if there is no preventive way [3,4]. China, United States, and Indonesia are country with the highest number of smoker in the world. Indonesia is also ranked the highest number of teenage smokers in the world [5]. They are 36.2% of men and 4.3% of female at 13-15 years old. Overall, there is 47.2% of teenage smokers addicted [6,7].

In the era of multimedia, the innovative interventions to promote smoking cessation behaviors is required to attract the teenager attention. Some behavior intervention such as edutainment and storytelling related to the health topic have provided to the teenager recently [8]. Manga comic, Japanese comic art, is the unique form of media combining the edutainment and storytelling which becomes teenagers´ favorite [9].

The educational comics of disease are varied, such as acquired immunodeficiency syndrome (AIDS) and sexual health, primary immunodeficiency, hepatitis B and tuberculosis [10]. Health education comic aims to improve the awareness of disease symptoms, prepare the patients (e.g. what has been expected from the medical procedure); assist the decision maker (e.g. deciding the treatment option); promote self-management from chronic conditions; or improve the understanding and accepting health condition [11]. Comic is not only to inform the fact, such as risk factor of the condition to the patients but also make them deal with the disease [12].

Comic as the media to give the information about smoking risk for the teenager was very limited. In this research, the researchers analyzed the influence of comic as the media to the knowledge and attitude of the smokers in the various levels of smoking behavior.

## Methods

**Study area:** this research was conducted in the Pontianak City, which is one of 14 cities in the province of West Kalimantan. Pontianak City has a population of 607,021 persons, and the largest city in the province. It is well planned, multicultural, cosmopolitan and the healthiest city in the province. It has established local regulations on non-smoking areas since 2010. But 14.7% of its teens are regular smokers, and surrounded by social environments that support these unhealthy behaviors.

**Study design:** this was an experimental study.

**Study population:** the population of the study was the students of junior high school in Pontianak City. They were 35,639 students aged 13-15 years old. Population data was obtained from basic education data published by the Ministry of Education and Culture of the Republic of Indonesia.

**Sample size:** this study took 1000 students as the sample.

**Sample technique:** a multistage sampling was employed. The sample was chosen from 4 different kind of schools, they were public junior high school, private junior high school, Islamic private junior high school, and Christian or Catholic private junior high school. The students were chosen randomly in those schools. The students who fit the requirements of this research were chosen to be the sample of this research.

**Study instruments:** a structured self-administered questionnaire with the following sections: sociodemographic, opinion toward comic, knowledge, attitude and practice was employed.

**Data collection method:** the questionnaire was pretested in one of the school in the study area that was not selected for the study. The questionnaires were then analyzed and changes were made based on the feedback received. Thereafter, ten trained data collectors were assigned to each of the selected districts to administer the questionnaire. They sought sociodemographic data and sections sought information on the opinion, knowledge, attitudes and practices about smoking cessation. The variables; knowledge, attitudes and practices were graded on a scale into five domains, a point was scored for correct.

**Study experiment:** this research experiment used manga style comic with color pages. The title was “Cool Teen Smoke-Free”. The comic content was about the harmful effect of smoking which were explained in 27 pages. The contents were: a) definition of smoking behavior; b) type of smoker (active and passive smoker); c) harmful chemical in cigarettes; d) diseases of smoking; e) quit-smoking tips.

**Study analysis:** data was entered, cleaned and analyzed using Microsoft Office Excel and Predictive Analytics SoftWare (PASW) Statistic version 18. Univariate analysis was performed for frequencies, means and proportions of sociodemographic characteristics of respondents and knowledge, attitudes, and practices (KAP) of respondents towards smoking cessation. Independent T-test (2-tailed) was used, as appropriate, to evaluate for significance of differences in responses between smoking status and knowledge and attitude of respondent towards smoking cessation and differences between social environment and knowledge and attitude of respondents toward smoking cessation. A p value of <0.05 was considered to be significant.

**Ethical clearance:** approval was obtained from the Research and Ethics Committee of Universitas Muhammadiyah Pontianak, Pontianak, Indonesia. Informed consent of the participants in the study were obtained and respondents were assured of confidentiality of information supplied.

## Results

This research aimed to find out the characteristic and attitude of teenage smoking in Pontianak. The data was collected according to the sex, experience after being educated, smokers´ family, smokers´ teacher, smokers´ friends, and smokers´ experience about smoking.

A total of 1,000 respondents were interviewed. According to Table 1, sociodemographic characteristics of respondents showed that the distribution of sex was 56% male and 44% female. It showed the prevalence of the respondents whose family are smokers. First, they were 35% respondents whose father smoke. Second, they were 5% respondents whose grandfather smoke. Third, they were 2% respondents whose family smoke. Furthermore, it showed teachers smoke at school. They were 32% respondents stated that some teachers are smokers, and even 2% respondents stated that most of their teachers are smokers. It can be seen about smoking status. They were 12% respondents who declared themselves as current smokers, 17% respondents smoked 1 cigarette and 13% respondents smoked less than 1 cigarette.

**Table 1:** frequency distribution of respondents according to personal smoking status and social environment smoking status

Sex	Frequency (N)	Percent (%)
Male	560	56
Female	440	44
**Family of smoker**		
No one	349	35
Smoker father	402	40
Smoker grandfather	48	5
Smoker sibling	55	6
Two of them are smoker	127	13
All smokers	19	2
**Smoker teacher**		
No one	334	33
A little	311	31
Some	322	32
Most of them	20	2
All	13	1
**Smoker status**		
Current smoker	119	12
Not a smoker	881	86
**Number of cigarettes per day**		
1-3 sticks	73	7
4-6 sticks	27	3
>6 sticks	19	2
Never	881	86

Table 2 shows the respondents´ who had been educated, had mean score of 14.1 with standard deviation of 1.7 in knowledge. Otherwise, the respondents who had not been educated had mean score of 13.8 with standard deviation of 1.9 in knowledge. The mean score of respondents who had been educated was higher than the respondents who had not been educated. This score was statistically significant (p<0.05). The mean score in attitude of the respondents who had been educated was 54.8, which was significantly different to the respondents who had not been educated (mean score 54.4). The mean score of two group respondents was not significantly different (p>0.05).

**Table 2:** comparison of knowledge and attitudes of respondent toward smoking cessation based on their anti-smoking education experience

Variables	Mean score	Standard deviation	P value
**Knowledge**			
Educated	14.1	1.7	0.017 *
Not educated	13.8	1.9	
**Attitude**			
Educated	54.8	3.8	0.106
Non-educated	54.3	4.1	

Table 3 shows there was no a significant difference (p value >0.05) in the mean score of knowledge between the respondents who had smoker families (mean score = 13.9) and the respondents who did not have smoker families (mean score = 14.1). In attitude aspect, the mean score of the respondents who had smoker families was 54.3 and the respondents who did not have smoker families´ was 55.0. So, there was significantly different in mean score of attitude (p value<0.05).

**Table 3:** comparison of knowledge and attitudes of respondent toward smoking cessation based on their smoker families

Variables	Mean	Standard deviation	P value
**Knowledge**			
Non-smoker families	14.1	1.7	0.34
Smoker families	13.9	1.9	
**Attitude**			
Non-smoker families	55	3.5	0.02*
Smoker families	54.3	4.1	

Analyze showed the impact of comics on the knowledge of respondents at various levels of smoking status. In fact, there was significant difference in the mean score of knowledge at smoking status (p value< 0.05). And, the impact of comics on the attitude of respondents at various levels of smoking status was significantly different (p value < 0.05). The mean score showed that the knowledge and attitude of respondents who did not smoke were higher than the knowledge and attitude of smoker respondents (Table 4).

**Table 4:** comparison of knowledge and attitudes of respondent toward smoking cessation based on their smoking status

Variables	Mean	Standard deviation	P value
**Knowledge**			
Smoker	13.6	2.1	0.03*
Non-smoker	14	1.8	
**Attitude**			
Smoker	51.2	5.5	0.00*
Non-smoker	55	3.4	

**Figure 1:**
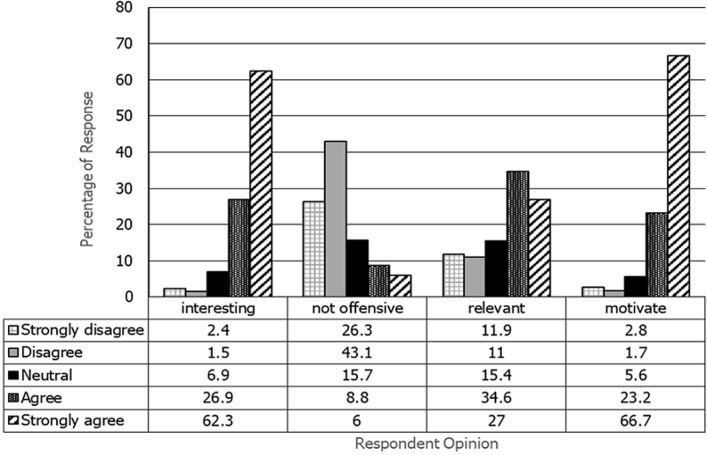
frequency distribution of respondents according to opinions about the comics design

There were 62.3% respondents strongly agreed that the material presented in the comic interesting. More than 69% of respondents stated that the story in the comic did not offend. Respondents also argued that the stories in comics were relevant to their conditions and background (51.6%). They also strongly agreed (66.7%) that comic content could prevent people to smoke (Figure 1).

## Discussion

As a communication medium, comics are associated with teenagers. However, last research suggests there may be benefits for health education. The comics were largely used to raise awareness about an issue and facilitate behavior change [13,14]. Other studies were proving the effect of comic for different groups and different health concerns, such as schistosomiasis prevention in school children in China [15]; stigma reduction and improved knowledge and attitudes toward lymphatic filariasis patients in Egypt [13]; cervical cancer screening promotion in women in South Africa [16]; and prostate cancer screening promotion in older men in Canada [17].

In this research, comics were designed and tested for the prevention and cessation of smoking. There is a difference in the effectiveness of information in several audience groups. The information about the harmful of smoking through comic is difficult to be accepted by the smokers. This comic is potential to convey the facts about disease of smoking and affect the audiences´ psychology. Some smokers are not ready to accept the fact that their smoking behavior is harmful to their health status. Comic help the patients to deal with the fear, anxiety, hope, and problem that they might be faced [11].

Respondents who have the intention to quit smoking have the bigger chance than the respondents who do not want to quit smoking [18]. It showed that motivating the smokers to think about smoking cessation by improving their awareness about the important of quitting to smoke through education is needed. It is also needed to motivate the smoker to quit smoking if they have tried to quit smoking but they failed [19]. This research aims to create innovative ways to providing the positive health message using the popular media. Comic education can be an innovative way to raise awareness about the importance of quitting smoking.

This research also found the fact that the effect of comic on audience knowledge (students) are influenced by social context. The students are surrounded by the smokers, such as smoker father, smoker brother, and smoker friend [20,21]. The attitude of teenage smokers are often related to the friendship. The popular classmates tend to smoke at school where is considered as normative place [22]. The popular students are more likely to be “trendsetters” for new behaviors, including smoking [23]. While parents are more influential in teenager, peers become the main source of influence in later adolescence through processes known as peer selection and peer influence [24].

Knowledge of risks and benefits of health behaviors associated with lifestyle are the prerequisites of performing a behavior. If people lack relevant knowledge, they will not accept reasons for enduring difficulties associated with that behavior. Comic as media are capable of providing information in the shortest possible time and maximum efficiency, and have a huge impact on knowledge, literacy, and attitudes of their audience [25].

There are several things that must be considered, when using comics as health education medium. Comics have a stigma as a medium for children and lower educated/lower income adult audiences [14]. Comics have also been reported as a medium which sex-stereotypes women [26]. Therefore, it should be considered when making health-promoting comics, they may need to be targeted and tailored to audience. This should also be considered if health-promoting comics are produced, as there should be multi-cultural, and even multi-spiritual, cast of characters, to appeal to all different types of groups. With rapid growth of modern media, including internet and mobile phone, and increasing number of people with access to such media, use of these media seems necessary to encourage healthy behavior among people. The strength of these media is in educating large numbers of people at low cost, compared to print media and face-to-face intervention [27]. So, in the future comics will no longer be printed, but will be shared through the internet or social media to be more efficient and affect more people.

## Conclusion

Based on the results and discussion, it can be concluded that comic is more effective in increasing the knowledge of adolescents who have anti-smoking training. This comic is also more effective in increasing the knowledge and attitudes of adolescents who do not have smoker family compared to adolescents who have smoker family. This comic is also more effective in increasing the knowledge and attitudes of non-smoker adolescents compared to smoker adolescents. It can be concluded that there is the impact of the comic as the media to the students' knowledge and behavior. In order to improve the efficiency of health education, it is better to use media to make the students interesting, such as comic. Comic can be used as learning media at school. The public health office can also build the cooperation with the schools to make the health promotion program, especially the harmful of smoking and they also have monitoring the program. Then, the school can have counselling program to help the students to quit smoking.

### What is known about this topic

- Tobacco smoking is one of the main killers in the world and Indonesia;
- The prevalence of youth tobacco smokers continues to increase in poor and middle-income economies countries;
- The cause of this prevalence increase is the lack of specific youth tobacco smoker interventions.

### What this study adds

- Smokers exist in youth age groups, they smoke 1-6 cigarettes regularly every day, even a small percentage smoke more than 6 cigarettes;
- Anti-smoking training and education experiences affect the level of knowledge after intervention by comic; smoking status also affects the level of knowledge after intervention; so the effectiveness of comics as a health education media for smoking cessation is influenced by smoking status and anti-smoking training experience of youth;
- Comic is an interesting health education media, because it consists of composition of colors, pictures and stories; the audience believes that the messages and stories conveyed through comic media are also relevant to the lives of youth, they also feel the message given motivates them to stay away from tobacco cigarettes.
